# Correspondence

**Published:** 1881-07

**Authors:** 


### CORRESPONDENCE.

Editor Dental Register:—Here is a second extract from a paper on fillings, read before the Illinois State Dental Society. Use it as your judgment may dictate, pruning it of any errors detrimental to the dental profession, that younger members be not misled.

Gutta percha in all its forms, when used as a filling is but a temporary stopping in my hands.

Amalgam fillings fail from expansion, contraction, improper insertion, improper finish and oxidation, and no matter how carefully instructed or diligent to learn the principles governing these, the operator is just as liable to failure where he needs the effect of this training, as he is to have a better filling where he is indifferent as to the result, and the novice is at still greater disadvantage.

Gold fillings fail from improper preparation of the cavity, want of adaptation to the walls, moving the margins in consolidation, roughening the edges and surface of the tooth around the filling when finishing, with a failure to properly polish this surface, especially at the cervix, allowing softened portions of enamel to remain difficult of access and sensitive to the excavator, risking a failure rather than a temporarily disagreeable impression, an improper finish of the occluding surface, the insertion of a gold filling where it is contra indicated, too severe malleting, wedging a piece of annealed gold between a wall and the main body of filling, to repair a defect after the gold is consolidated, causing undue pressure.

All the causes herein enumerated may be modified, and frequently avoided by careful manipulation and studious attention to details. I am aware, however, that there are many operators whose conceit and inaptitude in manipulative ability, preclude the possibility of success in the finer operations of operative dentistry; or perhaps, better say the converse of diligence prevents success.

I am aware too, that there are many sensitive natures who have the keenness of foresight, pliability for adaptation, latent skill and tact, sense of honor and ambition, to bless us by their advice and society, but who are too modest to attempt any public display for fear of criticism, and can not openly canvass the operations, essays, and conduct of those who care less for such judgment. We should encourage these by the hand of fellowship, enabling them to stem the tide and make an effort, despite forebodings and much perspiring. But besides all this, we owe it to our society and fellowmen beyond this narrower circle, to give whatever data we think we have, though humbly in broken sentences, and sometimes in imperfect clinics.

I have, at times, been astonished to find men who express themselves in the finest dicta, the principles of practical operations, almost totally incompetent to carry them out.

On the contrary, I have found men who would tremble, and alternately perspire and chill while in their seats, at the thought of attempting to speak in open society, and who, when they did speak, uttered broken sentences, saying almost totally the opposite of what they intended, becoming disgusted with themselves for making the attempt at all, not only capable of carrying out the principles enunciated by others, but were themselves astounded to find that they were really good operators.

Some of the best fillings I have seen, and these of gold, were from the hands of men who had passed through the trying ordeal of learning to talk in open society. Every member should be encouraged to emulation, and none discourged from the attempt. As gold has been attacked many times during the past few years, and the cheaper fillings bolstered by many a pen, let us see if there are not some redeeming features about practical reasons for our failures with gold. Some of these have been urged upon your attention and been disregarded. One of these is the improper preparation of the cavity. The literature of this society is replete with information on this subject, yet I wish to again say a few words here. I repeat, it seems to me impossible to properly prepare a cavity without first adjusting the rubber dam. Excavating is less painful after the cavity is dry—the walls and recesses are more readily disclosed, and in many cases the supposition of a pulp exposure is dispelled. I am satisfied that the truth has not been sufficiently impressed on our minds that the cervical wall should be excavated until the margin is solid, smooth, and free from a prominent ridge. There are softened lines of enamel near the cervix which extend laterally from cavities in proximal sufaces, which demand removal for success. Opportunites for seeing operations at the hands of another should not be lost, if we would have points of inestimable value discover themselves to us. In the clinics, follow the operator from beginning to end, and thus prove a patient student. In very large cavities fill the main portion with cement, and while pliable, make with a blunt excavator or old plugger, the size you wish, retaining pits in the cement, and after hardening, remove any protruding surplus obstructing the view and partially depend on these to anchor the filling. In many cases these retainers will be sufficient.

Many dentists commence filling by introducing pieces of gold too large for perfect adaptation to the walls. The edges should be true and smooth, but if a larger pellet is placed against the wall than can be made solid, an attempt will be made to condense it by undue pressure towards the face of the tooth and thus crush the margin, inviting certain decay; or, if the gold is not condensed against the floor or wall, in finishing there will be left a defect for future trouble. It frequently happens that a larger piece of gold than is suitable, is forced into a groove or pit, and the wall fractured or broken, and the tooth afterwards decays at that point. Another, and one of the most prolific causes of failure of gold fillings, is the finishing or want of true finish to the fillings and the enamel surface. In filing, great care should be taken not to mar the tooth, and to avoid a possible roughening of enamel about the filling. A thorough polish is of great benefit; one minute is not enough—five, ten, twenty—an hour—according to circumstances. You will become weary, and *then* your patient sometimes complain; be faithful and honest, and if you have been kind, he will come to you again with cheer. I believe there is more in the preservative effect in those fillings which are water-tight from leaving the enamel untouched, than from all the chemical and electro-chemical theories heard of. That the margins of the tooth around gold fillings in proximal cavities can be finished without marring the polished enamel, I know. But no careless, impatient dentist, who cares for the gain only, is capable of learning how it is done. Gold is the best material yet found for the *great majority* of cases, and no other filling material will save them equally well, as the expert must learn how to finish a filling of gold, so the bungler must rise to an immensely higher plane to be able to properly fill a cavity with gold.

Fillings that are too full and approach to near the pulp, will in the close approximation to that organ, by continued strokes, eventually cause inflammation and a deposit of "calcific granules" in its substance, and, assisted by thermal changes, result in its devitalization.

When the filling is too full at the grinding surface, we have the condition of an undue closure against it, and, unless the unfilled organ occluding the one filled, is worn away twice the amount of those without fillings, it will act as a constant source of neuralgia, and a constant reminder of the one-fourth million necessitous occlusions to keep up a year's mortal existence. The effect of the general wearing away of the teeth is not a release of the pressure on fillings, because metals, such as gold and amalgam are hardened on their hammered surfaces, and the oftner they are occluded the harder their surfaces become, until in the case of malleable amalgam and gold, the filling must break asunder, the walls of the tooth yield, or the tooth take on inflammation. Therefore, gold and amalgam fillings should be ground down, when they occlude an antagonist, at least, once a year. Under this constant physico-dental malleting the gold expands laterally, and sometimes causes pressure, resulting in dentine or enamel disintegration. If this pressure is not removed, so that the teeth will close evenly, the pericemental membrane will become thickened, and in the case of a tooth with a live pulp, produce a calcific deposit in that organ or upon the root, or in the case of a pulpless tooth, take on inflammation, which becoming chronic, will result in a fisula, with a constant flow of pus.

WATKINS, N. Y., May 25, 1881.

EDITOR DENTAL REGISTER:—Dear Sir:—Please excuse me for writing to you, but as I am in difficulty, I want advice.

I have made several alluminium plates during the last year, and with some have had excellent success, but with two partial plates made more recently (and the only partial ones I have made) there is a complaint of acidity in the mouth; the plates fit nicely, and no bearing upon any of the teeth. Some of the full sets had the teeth backed with rubber and some with celluloid. The partial were backed with celluloid.

Can you give me any information as to the cause of this trouble? I heard nothing of any similar trouble while at Ann Arbor.

Am sorry to find this difficulty, as I intended to use it quite extensively in place of rubber, which I do not like.

If you can give me any information as to the cause, I should be pleased to hear from you. As ever yours,

O. S. VOAK.

In reply to the above, Dr. W. H. Dorrance, of Ann Arbor, Mich., says:

"In reference, etc., to two partial cases of swaged alluminium base plates, "there is a complaint of acidity in the mouth," and inquires the cause.

Without doubt it is the result of galvanic action, arising from one of the following sources, viz:

1st. Connection with gold or other metallic fillings in the natural teeth, either by contiguity or through the medium of particles of food or the saliva.

2d. Alloys used as solders, or

3d. Impure or alloyed plate.

Some of the white alloys of alluminium and base metal are exceedingly suceptible to such action in the presence of the fluids of the mouth.

It is not at all likely that a pure alluminium plate, without the presence of any other metal, would be affected by any of the oral secretions, even if not normal, but the large use of pickles and salines, or the use of chloro-hydric acid in any form would effect the metal."

*Editor Dental Register:—*

DEAR SIR: For some eight or ten years the Illinois State Dental Society has been trying to get a law passed by our legislature for the regulation of the practice of dentistry,—or "to secure the better education of dentists." But year after year, for one reason and another, the committee having the matter in charge failed to succeed in procuring the passage of this law. During the early part of the present session of our legislature, the Chicago Dental Society appointed a committee of three, consisting of Drs. Harlan, Brophy and Talbot, to co-operate in this matter with the committee of the State Society. These gentlemen entered upon the discharge of their duty with the zeal and energy characteristic of men imbued with a determination to succeed. It was soon found, however, that while this committee was anxious to co-operate with the State Dental Society, that organization did not evince an over-weening desire to co-operate with the committee of the Chicago Dental Society.

Nothing daunted, however, at the cold shoulder presented by the committee of the State Society, the gentlemen from Chicago persevered, and left no stone unturned to secure the object in view. And although they met with very strong opposition in the legislature—the bill having been defeated both in the House and Senate during the present session—they succeeded in securing a rehearing in both houses and the subsequent passage of the bill, which is as follows:

*A Law to insure the better Education of Practitioners of Dental Surgery, and to regulate the practice of Dentistry in the State of Illinois.*

---

SECTION 1. *Be it enacted by the People of the State of Illinois, represented in the General Assemby,* That it shall be unlawful for any person who is not at the time of the passage of this act engaged in the practice of dentistry in this State to commence such practice, unless such person shall have received a diploma from the faculty of some reputable dental college, duly authorized by the laws of this State, or of some other of the United States, or by the laws of some foreign country, in which college or colleges there was at the time of the issue of such diploma annually delivered a full course of lectures and instructions in dental surgery: *Provided*, that the person removing into this State, who shall have been for a period of ten years prior to such removal, a practicing dentist, and: *Provided. also*, that any person holding the diploma of doctor of medicine from any reputable medical college, shall be entitled to practice dentistry in this State upon obtaining a license for the purpose as hereinafter provided; and nothing in this act shall be construed to prohibit any physican or surgeon from extracting teeth.

§ 2. A board of examiners, to consist of five practicing dentists, is hereby created, whose duty it shall be to carry out the purposes and enforce the provisions of this act. The members of said board shall be appointed by the Governor.

The term for which the members of said board shall hold their offices shall be five years, *except*, that the members of the board first to be appointed under this act, shall hold their offices for the term of one, two, three, four and five years respectively, and until their successors shall be duly appointed.

In case of a vacancy occurring in said board, such vacancy shall be filled by the Governor.

§ 3. Said board shall choose one of its members president, and one the secretary thereof, and it shall meet at least once in each year, aud as much oftener, and at such time and places, as it may deem necessary. A majority of said board shall at all times constitute a quorum, and the proceedings thereof shall at all reasonable times be open to public inspection.

§ 4. It shall be the duty of every person who is engaged in the practice of dentistry in this State, within six months from the date of the passage of this act, to cause his or her name and residence or place of business to be registered with said board of examiners, who shall keep a book for that purpose; and every person who shall so register with said board as a practitioner of dentistry, may continue to practice the same as such, without incurring any of the liabilities or penalties provided in this act.

§ 5. No person whose name is not registered on the books of said board as a regular practitioner of dentistry, within the time prescribed in the preceding section, shall be permitted to practice dentistry in this State until such person shall have been duly examined by said board and regularly licensed in accordance with the provisions of this act.

§ 6. Any and all persons, who shall so desire, may appear before said board at any of its regular meetings and be examined with reference to their knowledge and skill in dental surgery, and if the examination of any such person or persons shall prove satisfactory to said board, the board of examiners shall issue to such persons, as they shall find from such examination to possess the requisite qualifications, a license to practice dentistry in accordance with the provisions of this act. But said board shall at all times issue a license to any regular graduate of any reputable dental college without examination, upon the payment by such graduate to the said board of a fee of one dollar. All licenses issued by said board shall be signed by the members thereof and be attested by its president and secretary; and such evidence shall be *prima facie* evidence of the right of the holder to practice dentistry in the State of Illinois.

§ 7. Any member of said board may issue a temporary license to any applicant, upon the presentation by such applicant of the evidence of the necessary qualifications, to practice dentistry, and such temporary license shall remain in force until the next regular meeting of said board occurring after the date of such temporary license, and no longer.

§ 8. Any person who shall violate any of the provisions of this act shall be liable to prosecution, before any court of competent jurisdiction, upon information or by indictment, and upon conviction may be fined not less than twenty-five dollars, nor more than fifty dollars, for each and every offense. All fines recovered under this act shall be paid into the common school fund of the county in which such conviction takes place.

§ 9. In order to provide the means for carrying out and maintaining the provisions of this act, the said board of examiners may charge each person applying to or appearing before them for examination for license to practice dentistry, a fee of two dollars, and out of the funds coming into the possession of the board from the fees so charged, the members of said board may receive as compensation the sum of five dollars for each day actually engaged in the duties of their office, and all legitimate and necessary expenses incurred in attending the meetings of said board. Said expenses shall be paid from the fees and penalties received by the board under the provisions of this act. And no part of the salary or other expenses of the board shall ever be paid out of the State Treasury. All moneys received in excess of said per diem allowance, and other expenses above provided for, shall be held by the secretary of said board as a special fund for meeting the expenses of said board, he giving such bond as the board shall from time to time direct. And said board shall make an annual report of its proceedings to the Governor, by the fifteenth of December of each year, together with an account of all moneys received and disbursed by them, pursuant to this act.

§ 10. Any person who shall be licensed by said board to practice dentistry shall cause his or her license to be registered with the county clerk of any county or counties in which such person may desire to engage in the practice of dentistry, and the county clerks of the several counties in this State shall charge for registering such license a fee of twenty-five cents for each registration.

Any failure, neglect or refusal on the part of any person holding such license to register the same with the county clerk as above directed for a period of six months shall work a forfeiture of the license, and no license when once forfeited, shall be restored, except upon the payment to the said board of examiners the sum of twenty-five dollars, as a penalty for such neglect, failure or refusal."

And henceforth it is to be hoped that so-called dentists, who, on account of their inability to pass an examination, have been driven from other states, will not be allowed to rendezvous in the state of Illinois for the practice of their quackery.

ILLINOIS.

